# Factors associated with delayed presentation to healthcare facilities for Lassa fever cases, Nigeria 2019: a retrospective cohort study

**DOI:** 10.1186/s12879-021-05822-4

**Published:** 2021-02-04

**Authors:** Nastassya L. Chandra, Hikaru Bolt, Chioma Dan-Nwafor, Oladipupo Ipadeola, Elsie Ilori, Geoffrey Namara, Adebola T. Olayinka, Winifred Ukponu, Akanimo Iniobong, Michael Amedu, Adejoke Akano, Kachikwulu O. Akabike, Uwaifiokun Okhuarobo, Stephen Fagbemi, Emeka Sampson, Sophie Newitt, Neville Q. Verlander, Daniel G. Bausch, Olivier le Polain de Waroux, Chikwe Ihekweazu

**Affiliations:** 1 UK Field Epidemiology Training Programme, Public Health England, London, UK; 2 UK Public Health Rapid Support Team - Public Health England/London School of Hygiene & Tropical Medicine, London, UK; 3 Nigeria Centre for Disease Control, Abuja, Nigeria; 4 World Health Organization, Abuja, Nigeria; 5 Georgetown University, Centre for Global Health Practice and Impact, Abuja, Nigeria; 6 Edo State Ministry of Health, Edo, Nigeria; 7 Ondo State Ministry of Health, Ondo, Nigeria; 8 Ebonyi State Ministry of Health, Ebonyi, Nigeria; 9 Public Health England, National Infection Service, London, UK

**Keywords:** Lassa fever, Nigeria, Surveillance, Epidemiology, Delayed presentation, Healthcare, Retrospective cohort study

## Abstract

**Background:**

Large outbreaks of Lassa fever (LF) occur annually in Nigeria. The case fatality rate among hospitalised cases is ~ 20%. The antiviral drug ribavirin along with supportive care and rehydration are the recommended treatments but must be administered early (within 6 days of symptom onset) for optimal results. We aimed to identify factors associated with late presentation of LF cases to a healthcare facility to inform interventions.

**Methods:**

We undertook a retrospective cohort study of all laboratory confirmed LF cases reported in Nigeria from December 2018 to April 2019. We performed descriptive epidemiology and a univariate Cox proportional-hazards regression analysis to investigate the effect of clinical (symptom severity), epidemiological (age, sex, education, occupation, residential State) and exposure (travel, attendance at funeral, exposure to rodents or confirmed case) factors on time to presentation.

**Results:**

Of 389 cases, median presentation time was 6 days (IQR 4–10 days), with 53% attending within 6 days. There were no differences in presentation times by sex but differences were noted by age-group; 60+ year-olds had the longest delays while 13–17 year-olds had the shortest. By sex and age, there were differences seen among the younger ages, with 0–4-year-old females presenting earlier than males (4 days and 73% vs. 10 days and 30%). For 5–12 and 13–17 year-olds, males presented sooner than females (males: 5 days, 65% and 3 days, 85% vs. females: 6 days, 50% and 5 days, 61%, respectively). Presentation times differed across occupations 4.5–9 days and 20–60%, transporters (people who drive informal public transport vehicles) had the longest delays. Other data were limited (41–95% missing). However, the Cox regression showed no factors were statistically associated with longer presentation time.

**Conclusions:**

Whilst we observed important differences in presentation delays across factors, our sample size was insufficient to show any statistically significant differences that might exist. However, almost half of cases presented after 6 days of onset, highlighting the need for more accurate and complete surveillance data to determine if there is a systemic or specific cause for delays, so to inform, monitor and evaluate public health strategies and improve outcomes.

## Background

Lassa fever (LF) infection is a viral haemorrhagic fever (VHF) caused by a single stranded RNA virus belonging to the genus *Mammarenavirus. Lassa mammarenavirus* is transmitted to humans via contact with food or household items contaminated with rodent urine or faeces, particularly a rodent of the genus *Mastomys*, commonly known as the multimammate rat. *Lassa mammarenavirus* may also be spread between humans through direct contact with the blood, urine, faeces, or other bodily secretions of an infected person [1, 2]. However, secondary attack rates are generally less than 5% [1–3].

Lassa fever is endemic in Nigeria, with a seasonal pattern, peaking in the dry season (November to April) [3]. Recent years have seen an increase in the incidence of LF reported in Nigeria [3, 4]. Between January and May 2019, there were 578 confirmed cases in 21 states with a case fatality rate of approximately 22%, surpassing the total case counts in 2017 and 2018 [5–7].

The incubation period of LF ranges from 2 to 21 days [2]. The onset of the disease, when symptomatic, is usually gradual, starting with non-specific symptoms then developing to more severe symptoms (including haemorrhage) after a week or so. The case fatality rate (CFR) among hospitalised cases is approximately 20%, but mild or asymptomatic infection is common [1–3].

Early treatment is considered vital in LF and delays in presentation to healthcare facilities may impact survival. Current recommended treatment is largely supportive (rehydration and symptomatic treatment). Although controversy exists over its efficacy, intravenous administration of the antiviral drug ribavirin has long been considered the standard of care, with one study showing the most impact on survival if given within the first 6 days after symptom onset [1–3, 8–10].

Investigation of the 2018–19 LF outbreak in Nigeria showed a higher CFR among people presenting later to healthcare facilities, suggesting that time to presentation and treatment may indeed be a key intervention point to reduce mortality. Studies on tuberculosis and trypanosomiasis in Africa have reported that delays in presentation to a healthcare facility were associated with many factors, including sex, age, rurality, and socio-economic status [11–13]. Informed by these studies and the three delays model [14, 15], we hypothesised that similar factors play an important role in delayed presentation time for people with LF.

To inform public health interventions including targeted health education, risk communication and preventative measures, it is important to identify factors that are associated with cases presenting later to healthcare facilities. We aimed to examine whether there are demographic, exposure, or clinical factors associated with delayed presentation of people with LF to a healthcare facility in Nigeria.

## Methods

We undertook a retrospective cohort study of all laboratory confirmed LF cases reported through routine national outbreak surveillance data in Nigeria with onset dates between December 2018 and April 2019.

### Data collection and management

During this period, national routine surveillance for LF comprised data from laboratory, case management (clinical) and case investigation (epidemiological) forms. The forms captured demographic information (age, sex, residential address, occupation), date of admission, hospital name, clinical details (date of symptom onset, symptoms) and exposures (contact with known or suspected LF case, being part of a contact tracing list, history of travel, direct contact with rodents or rodent faeces and urine, participation in burial activity).

**Fig. 1:**
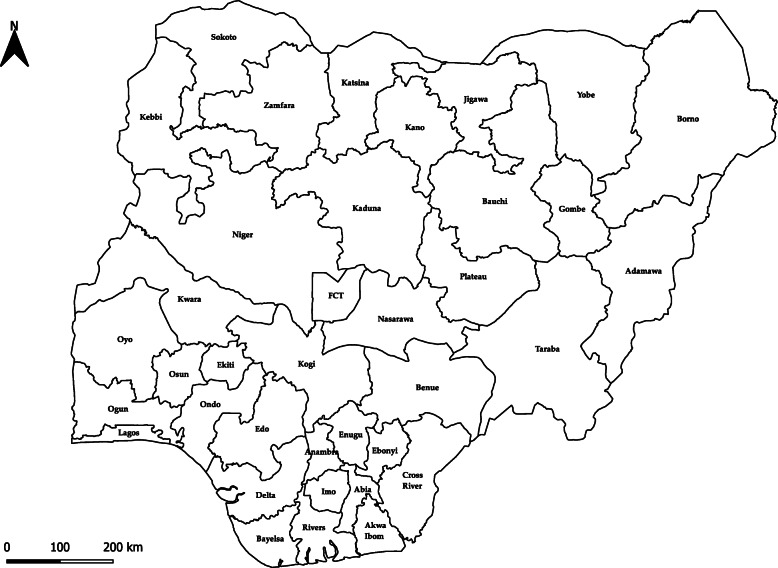
Map of Nigeria. Map produced by study authors on behalf of Nigeria Centre for Disease Control. FCT = Federal Capital Territory

The case management forms were completed at the healthcare facility that the case presented to. Specimen samples were tested at the laboratories at the Federal Teaching Hospital Abakaliki (FETHA), Irrua Specialist Teaching Hospital (ISTH), Lagos University Teaching Hospital (LUTH), Federal medical Centre Owo LF Laboratory and the National Reference Laboratory (NRL) Gaduwa. Average turnaround time of the diagnostic results is 48 h, ranging between 24 to 96 h. Turnaround time is largely dependent on the proximity of the state to the testing laboratory. The testing laboratory and a Local Government Area (LGA) Disease Surveillance and Notification Officer or State Epidemiologist entered the data into a line list and/or in the Surveillance Outbreak Response Management and Analysis System (SORMAS) platform (implemented in 12 states). An LGA is an administrative geographical division of a state. There are 774 LGAs across the 36 states of Nigeria and the Federal Capital Territory (Fig. 1) [16]. The Nigeria Centre for Disease Control managed and linked the data.

### Data cleaning and analysis

Data were de-duplicated and anonymised in Stata 14. Presentation time was defined as the difference, in days, between date of symptom onset and date of presentation at a healthcare facility. Considering biological plausibility, only those with a presentation time between 1 and 21 days were included.

Clinical symptoms were categorised as mild or severe based on LF literature [17, 18]. If a case had mild and severe symptoms, they would be categorised as severe if a case had only mild symptoms they would be categorised as mild. For example, headache, slight fever, general malaise and weakness would be categorised as a mild symptoms and haemorrhaging (in gums, eyes, or nose, as examples), respiratory distress, repeated vomiting, facial swelling, pain in the chest, back, and abdomen, and shock would be categorised as severe symptoms [17, 18]. Rurality of residence was based on the residential LGA mapped to a rurality index table (rural, semi-urban and urban) [19, 20]. Age data were grouped based on life stages (0–4, 5–12, 13–17, 18–25, 26–59 and 60+ year-olds). Sex and age-groups were looked at separately and together.

Malaria is highly prevalent in some areas of Nigeria and early symptoms of LF are similar (e.g. fever, headaches, vomiting) [21]. Understandably, people may incorrectly assume early symptoms of LF are those of malaria which may affect time to presentation. Malaria prevalence category was based on the findings of the Nigeria Malaria Indicator Survey (2015). We used the prevalence in children as a proxy for malaria prevalence in the cases’ state of residence [16].

To assess completeness, the proportion of missing data was calculated. To determine if missing data were completely at random, a mixed effects logistical regression for age-group and sex was undertaken.

We described the time to presentation (range, interquartile range, median time in days) and the number of cases that attended a healthcare facility within 6 days or less following symptom onset by demographic, clinical and exposure factors. We undertook a univariate analysis using a Cox Proportional-Hazards regression model to investigate the effect of each variable on the time to presentation. The Proportional Hazard assumption was tested on the basis of Schoenfeld residuals after fitting the Cox regression model.

Characteristics of people attending healthcare facilities in certain LGAs may be more similar e.g. a higher proportion of farmers in more rural LGAs. We accounted for LGA level clustering using a multi-level model to include the random effects of LGA to attending a healthcare facility.

For all models, 95% confidence intervals and *p*-values were calculated using the Wald test. A multivariable analysis was not performed following the univariate Cox Proportional-Hazards analysis, as no variables had a *p*-value of < 0.2, an a priori requirement.

### Ethics

Ethical committee clearance was provided to undertake this study which uses retrospective routine data collected as part of LF surveillance in Nigeria, further details provided below.

## Results

### Descriptive epidemiology

Of 499 confirmed LF cases in the dataset, 417 (84%) had complete dates for symptom onset and attendance at a healthcare facility. Of these, 389 (78% of all cases) had presentation dates between 1 and 21 days, and thus comprised our dataset for analysis.

The median time to presentation to a healthcare facility was 6 days (Inter quartile range [IQR] 4–10 days), with 53.4% attending in 6 days or less (Table 1). Males and females had the same median time to presentation (6 days).

**Table 1:** Lassa fever case characteristics and time to presentation, Nigeria 2019, (*n* = 389)

Characteristic	Stratum	Number of cases (%)	Number of cases attending in 6 or less days (%)	Median time to presentation (days)	Interquartile range of time to presentation (days)	Range of time to presentation (days)
**Case definition number of cases**	Laboratory Confirmed	389	208 (53.4)	6	4–10	1–21
**Sex**	Female	181 (46.5)	101 (55.8)	6	3–10	1–21
	Male	207 (53.2)	107 (51.7)	6	4–10	1–21
	Unknown	1 (0.3)	0	7	n/a	n/a
**Age group (years)**	0–4	21 (5.4)	11 (52.4)	6	4–13	1–16
	5–12	35 (9.0)	20 (57.1)	6	4–9	1–21
	13–17	31 (8.0)	22 (71.0)	5	2–8	1–21
	18–25	56 (14.4)	33 (58.9)	6	3–10	1–21
	26–59	214 (55.0)	107 (50.0)	6	4–10	1–21
	60+	31 (8.0)	14 (45.2)	7	3–10	1–14
	Unknown	1 (0.3)	1 (100)	4	n/a	n/a
**Age group (years) and Sex (*n*** **= 387)**						
**Males (*n*** **= 206)**	0–4	10 (4.9)	3 (30.0)	10	5–15	3–16
	5–12	17 (8.3)	11 (64.7)	5	4–8	1–20
	13–17	13 (6.3)	11 (84.6)	3	3–6	2–21
	18–25	27 (13.1)	16 (59.3)	6	3–10	1–16
	26–59	123 (59.7)	59 (48.0)	7	5–10	1–21
	60+	16 (7.8)	6 (37.5)	7	2–10	1–14
**Females (*n*** **= 181)**	0–4	11 (6.1)	8 (72.7)	4	3–7	1–14
	5–12	18 (9.9)	9 (50)	6	4–10	1–21
	13–17	18 (9.9)	11 (61.1)	5	2–9	1–18
	18–25	29 (16)	17 (58.6)	6	3–12	1–21
	26–59	90 (49.7)	48 (53.3)	6	4–12	1–20
	60+	15 (8.3)	8 (53.3)	6	3–10	1–14
**Mortality status outcome**	Alive	324 (83.3)	175 (54.0)	6	4–10	1–21
	Deceased	65 (16.7)	33 (50.8)	6	3–10	1–19
**State of residence**	Abia*	0	n/a	n/a	n/a	n/a
	Adamawa	0	n/a	n/a	n/a	n/a
	Akwa Ibom*	0	n/a	n/a	n/a	n/a
	Anambra*	0	n/a	n/a	n/a	n/a
	Bauchi	36 (9.3)	13 (36.1)	8	6–13	1–21
	Bayelsa*	0	n/a	n/a	n/a	n/a
	Benue	4 (1.0)	1 (25.0)	8	6–10	5–11
	Borno*	0	n/a	n/a	n/a	n/a
	Cross River	1 (0.3)	0	15	n/a	n/a
	Delta	2 (0.5)	0	13.5	13–14	13–14
	Ebonyi	33 (8.5)	14 (42.4)	7	4–10	1–14
	Edo	171 (44.0)	94 (55.0)	6	4–10	1–21
	Enugu	0	n/a	n/a	n/a	n/a
	Ekiti*	0	n/a	n/a	n/a	n/a
	Federal Capital Territory	1 (0.3)	0	11	n/a	n/a
	Gombe	1 (0.3)	1 (100)	4	n/a	n/a
	Imo	1 (0.3)	0	8	n/a	n/a
	Jigawa*	0	n/a	n/a	n/a	n/a
	Kaduna	3 (0.8)	2 (66.7)	5	3–20	3–20
	Kano	0	n/a	n/a	n/a	n/a
	Katsina*	0	n/a	n/a	n/a	n/a
	Kebbi	1 (0.3)	1 (100)	3	n/a	n/a
	Kogi	2 (0.5)	0	10	8–12	8–12
	Kwara	1 (0.3)	1 (100)	2	n/a	n/a
	Lagos*	0	n/a	n/a	n/a	n/a
	Nasarawa	2 (0.5)	0	14.5	8–21	8–21
	Niger*	0	n/a	n/a	n/a	n/a
	Ogun*	0	n/a	n/a	n/a	n/a
	Ondo	87 (22.4)	58 (66.7)	6	3–7	1–20
	Osun*	0	n/a	n/a	n/a	n/a
	Oyo	1 (0.3)	1 (100)	5	n/a	n/a
	Plateau	27 (6.9)	13 (48.2)	7	2–13	1–20
	Rivers	1 (0.3)	1 (100)	4	n/a	n/a
	Sokoto*	0	n/a	n/a	n/a	n/a
	Taraba	14 (3.6)	8 (57.1)	6	5–14	2–20
	Yobe*	0	n/a	n/a	n/a	n/a
	Zamfara*	0	n/a	n/a	n/a	n/a
**Rurality category (based on state of residence and information from state guidance)**	Rural	238 (61.2)	124 (52.1)	6	4–11	1–21
	Semi-urban	14 (3.6)	7 (50.0)	6.5	4–9	3–12
	Urban	134 (34.4)	75 (56.0)	6	4–9	1–20
	Unknown	3 (0.8)	2 (66.7)	4	1–15	1–15
**Malaria Prevalence category**	0–11%	4 (1.0)	1 (25.0)	8	6–10	4–12
	12–21%	298 (76.6)	166 (55.7)	6	4–10	1–21
	22–29%	3 (0.8)	2 (66.7)	4	2–15	2–15
	30–36%	62 (15.9)	27 (43.55)	7	4–10	1–21
	37–64%	22 (5.7)	12 (54.6)	7	5–11	2–20
**Education status**	Primary	20 (5.1)	10 (50.0)	6.5	5–9.5	3–20
	Secondary	32 (8.2)	19 (59.4)	5	3–9	1–15
	Tertiary	35 (9.0)	17 (48.6)	7	3–10	1–20
	Unknown	302 (77.6)	162 (53.6)	6	4–10	1–21
**Occupation group**	Artisan	11 (2.8)	6 (54.6)	6	4–11	2–15
	Business or Professional^a^	101 (26.0)	45 (44.6)	7	4–11	1–20
	Casual Labourer	1 (0.3)	0	7	n/a	n/a
	Farmer	35 (9.0)	21 (60.0)	6	4–10	1–19
	Health Worker	12 (3.1)	7 (58.3)	4.5	3.5–9.5	2–13
	Housewife	32 (8.2)	14 (43.8)	7	5–13	1–21
	Religious or Traditional^a^	6 (1.5)	2 (33.3)	7	5–7	3–10
	Student or Child or Apprentice	126 (32.4)	72 (57.1)	6	3–10	1–21
	Transporter	5 (1.3)	1 (20.0)	9	7–10	4–15
	Not working or Retired	10 (2.6)	4 (40.0)	8.5	3–12	1–14
	Unknown	50 (12.9)	36 (72.0)	6	3–7	1–21
**Symptoms**	Mild	123 (31.6)	78 (63.4)	6	4–8	1–21
	Severe	22 (5.7)	12 (54.6)	6	3–8	1–14
	Unknown	244 (62.7)	118 (48.4)	7	4–11	1–21
**First symptom**	Mild	167 (42.9)	83 (49.7)	7	4–11	1–21
	Severe	2 (0.5)	1 (50.0)	8	2–14	2–14
	Unknown	220 (56.6)	124 (56.4)	6	4–9.5	1–21
**Exposure to confirmed case**	No	87 (22.4)	43 (49.4)	7	4–9	1–20
	Yes	39 (10.0)	20 (51.3)	6	4–10	1–20
	Unknown	263 (67.6)	145 (55.1)	6	4–10	1–21
**Exposure to rodents**	No	8 (2.0)	2 (25.0)	9	6.5–16	3–20
	Yes	12 (3.1)	4 (33.3)	8	6–12	2–20
	Unknown	369 (94.9)	202 (54.7)	6	4–10	1–21
**Part of a contact tracing list**	No	124 (31.4)	57 (46.0)	7	4.5–11	1–21
	Yes	36 (9.8)	19 (52.8)	6	3.5–10	1–20
	Unknown	229 (58.8)	132 (57.6)	6	3–10	1–21
**History of travel**	No	279 (71.7)	148 (53.1)	6	4–10	1–21
	Yes	86 (22.1)	48 (55.8)	6	4–11	1–20
	Unknown	24 (6.2)	12 (50.0)	6.5	3–13.5	2–21
**Attended a funeral**	No	230 (59.1)	124 (53.9)	6	3–10	1–21
	Yes	1 (0.3)	0	11	n/a	n/a
	Unknown	158 (40.6)	84 (53.2)	6	5–10	1–21
**Participated in a funeral (touched body)**	No	156 (40.1)	75 (48.1)	7	4–10	1–21
	Yes	4 (1.0)	2 (50.0)	6	2–10	2–13
	Unknown	229 (58.9)	131 (53.5)	6	4–10	1–21

Includes only those that have a non-missing value for the time to presentation variable and the characteristic and for presentation times between 1 and 21 days

^a^ Business or professional: A business person here refers to one who earns profit by supplying goods and services while a professional refers to someone with specified qualifications or expertise who gets paid for rendering services. Religious or Traditional: Traditional healers use indigenous plants, herbs and the root of plants to heal sick people while religious healer use prayers predominantly to heal sick people

*No information for this state has been reported in this dataset

By age-group only, median presentation time ranged between 5 and 7 days with 45.2 to 71.0% of cases presenting within 6 days of onset.

By sex, there were differences seen among the younger age-groups, with 0–4 year-old females presenting earlier than males (females: 4 days [IQR 3–7 days] and 73%, males: 10 days [IQR 5–15] and 30%). For 5–12 and 13–17 year-olds, males had a shorter median presentation time and higher proportion presenting within 6 days of onset compared to females (males aged 5–12 years: 5 days [IQR 4–8], 65% and males aged 13–17 years: 3 days [IQR 3–6], 85%; females aged 5–12 years: 6 days [IQR 3–7], 50% and females aged 13–17: 5 days [IQR 2–9], 61%).

Most cases in the dataset came from Edo (44.0%) and Ondo (22.4%) States, who had median presentation times of 6 days. The longest presentation times were seen in Cross River (15 days) and Nasarawa (14.5 days, [IQR 8–21]) but case numbers were small. Most cases resided in rural areas (61.2%). Approximately 50% of cases in each rurality index category attended within 6 days.

Cases living in areas with the lowest malaria prevalence category (0–11%) had the longest median presentation time of 8 days (IQR 6–10) and 25% (1 case) attended within 6 days.

By occupation, transporters (people who drive informal public transport vehicles) had the longest median presentation time (9 days [IQR 7–10]) closely followed by those not working/retired (8.5 days [IQR 3–12]).

Data for education status and symptoms were limited (77.9 and 62.7% missing). Exposure data was poorly completed (between 40.6 and 94.9% missing, with the exception of travel history, for which only 6.2% were missing). Where information was available for travel history, people who had a travel history had the same median time to presentation as those who did not have a history of travel (6 days).

### Missing data analysis

When comparing age groups for those with missing presentation time to those without missing presentation time, there was no statistical evidence of a difference (*p*-value 0.77). When comparing by sex, there was evidence of a difference, with females less likely to have missing presentation times than males (odds ratio 0.57, 95%CI 0.34–0.95, p-value 0.03).

### Univariate analysis

There was no statistically significant difference between any variable with regard to time to presentation to a healthcare facility (Table 2). The proportional hazard assumption was met for all variables at the 5% significant level.

**Table 2:** Cox univariate analysis of confirmed cases by demographic, clinical and exposure factors, Nigeria 2019, (*n* = 389)

Characteristic	Stratum	Hazard ratio	Crude 95% CI	Crude p-value	Proportional Hazard test of assumption
**Sex**	Male	Ref		0.97	0.28
	Female	1.10	0.82–1.23		
**Age (per year)**		1.00	0.99–1.01	0.95	0.05
**Age group (years)**	0–4	Ref		0.76	0.11
	5–12	1.08	0.60–1.89		
	13–17	1.25	0.71–2.21		
	18–25	0.91	0.55–1.53		
	26–59	0.97	0.61–1.53		
	60+	1.11	0.63–1.96		
**Mortality status**	Dead	Ref			
	Alive	0.97	0.73–1.28	0.81	0.45
**Rurality**	Rural	Ref		0.34	0.37
	Semi-urban	1.39	0.79–2.46		
	Urban	1.15	0.89–1.49		
**Malaria prevalence category**	0–11%	Ref		0.37	0.88
	12–21%	0.98	0.36–2.71		
	22–29%	4.15	0.74–23.44		
	30–36%	1.00	0.36–2.83		
	37–64%	0.85	0.28–2.64		
**Education status**	Primary	Ref		0.38	0.97
	Secondary	1.44	0.81–2.55		
	Tertiary	1.1	0.63–1.91		
**Occupation group**	Not working or retired	Ref		0.95	0.65
	Student or Child or Apprentice	1.13	0.57–2.23		
	Housewife	0.89	0.42–1.88		
	Farmer	1.21	0.58–2.54		
	Casual labourer	1.26	0.16–10.02		
	Transporter	0.91	0.30–2.72		
	Religious or Traditional^a^	1.45	0.51–4.10		
	Artisan	1.15	0.47–2.78		
	Business or Professional^a^	1.10	0.55–2.18		
	Health Worker	1.47	0.61–3.55		
**Symptom severity**	Mild	Ref		0.90	0.61
	Severe	1.03	0.65–1.63		
**Exposure to rodents**	No	Ref		0.69	0.82
	Yes	1.23	0.45–3.34		
**Exposure to confirmed case**	No	Ref		0.77	0.93
	Yes	0.94	0.64–1.38		
**History of Travel**	No	Ref		0.76	0.26
	Yes	1.04	0.81–1.34		
**Attended a funeral**	No	Ref		0.79	0.35
	Yes	0.65	0.09–4.68		
**Participated in a funeral (touched body)**	No	Ref		0.92	0.64
	Yes	1.05	0.38–2.90		

^a^ Business or professional: A business person here refers to one who earns profit by supplying goods and services while a professional refers to someone with specified qualifications or expertise who gets paid for rendering services. Religious or Traditional: Traditional healers use indigenous plants, herbs and the root of plants to heal sick people while religious healer use prayers predominantly to heal sick people

## Discussion

Multiple factors may contribute to a delayed presentation to a healthcare facility and seeking early treatment and care for LF. This increases the likelihood of severe illness and death. Our study showed that almost half of all confirmed cases presented to a healthcare facility 6 days after symptom onset, but we did not find any specific factors statistically associated with delayed presentation of confirmed cases. This suggests that there is need for more public health action and messaging around LF and seeking healthcare.

We found that particular groups have longer median presentation times or present less often within 6 days of symptom onset. Edo and Ondo states had a high number of cases captured within the surveillance system compared to the other states but did not have the longest presentations delays. The shorter presentation times in Edo and Ondo may be attributed to the historical high incidence of LF in these states leading to a higher index of suspicion and greater awareness among the population. Areas such as Nasarawa and Cross Rivers states, which had fewer cases but the longest presentation times, may benefit from more targeted risk communication and public health action. While the regression analysis did not show any factors associated with later presentation, it is important to recognise that almost half of the cases presented 6 days after symptom onset. There may be a systemic reason for cases to present later which may not be evident from this study.

The findings of the Cox regression are somewhat unexpected, as other studies investigating health seeking behaviours, delays in diagnostics and presentation for other infectious and parasitic diseases have found significant associations with various factors. Ukwaja et al., 2013, found that factors associated with increased presentation delay in Nigeria for tuberculosis cases were older age, distance to a health facility and urban residency [13]. However, a study by Hulland et al., 2019 showed that in Nigeria, 98% of the population at risk of VHFs had travel times to a health facility of under 2 h with exceptions of some states, so this may not be a factor for delayed presentation for LF [22]. In Zambia, Needham et al., 2001 found that there were significant socio-demographic factors associated with delay, including being female and only having an education up to the age of 9 years [11]. For Ebola disease, another VHF, Theocharopoulos et al., 2017, found that transportation time and age affected the time to admission for Ebola patients in Sierra Leone [23]. A common practice in Nigeria, particularly in Northern states, is for a woman to seek her husband’s consent before going out therefore not seeing an association with sex and time to presentation is unexpected [24].

We believe that our findings resulted in part from limitations of the surveillance data, including a lack of data from multiple years of outbreaks and high quality and complete epidemiological and exposure data. Education status was missing for almost 80% of the confirmed cases in our dataset, with no option provided for ‘no formal education’. Symptom data were missing for more than 60% of cases and exposure data was missing in up to 95% of cases. This limits the interpretation of our findings and potential interventions to be put into place. More years of surveillance data, collected in a consistent manner with higher completion rates for variables, may provide a clearer picture of factors associated with presentation delays, which may be important to inform public health messaging.

### Study limitations

Firstly, this analysis was restricted to confirmed case records that had completed symptom onset and healthcare attendance dates (78% of confirmed cases); this may increase the risk of selection bias limiting the generalisability of the analysis. We compared cases with missing presentation times by age and sex in a logistic regression. For age group, there was no significant difference between those with and without presentation time, suggesting that the dates may be missing completely at random. However, for sex, there was a statistical difference, with males more likely to have missing presentation dates. Therefore, data may not be missing completely at random and may have introduced bias into our results.

Secondly, the completion of symptom onset dates varied across different LGA areas and/or health facilities. We decided to exclude dates where presentation time was 0 days or greater than 21 days. This may have excluded true data, but it is unlikely given the mild symptoms at onset and the likelihood of death or resolution after 21 days. A presentation time of 0 could have occurred if the case had known LF exposures or were part of a contact tracing list; but these fields were poorly completed.

Thirdly, underreporting in certain states may be an issue, with several states reporting little or no cases, which may not be a true indication of the incidence.

Finally, residual confounding could occur as only a few variables are currently collected for establishing presentation delay, which may limit interpretation of factors associated with delays.

### Future work

To generate hypotheses, important gaps in LF surveillance data need to be addressed across all states of Nigeria. This requires a coordinated effort nationally and regionally. Detailed and complete information needs to be captured for clinical and exposure factors. This work is currently ongoing in Nigeria with the complete roll-out and use of the Surveillance Outbreak Response Management and Analysis System (SORMAS), which was partially rolled out at the time of this study (12 states). Further details around SORMAS are found elsewhere [25]. With the wider use of SORMAS, the linked electronic system allows improved data completion, removes some of the traditional paper-based forms, improving the data quality and removing duplication of efforts. It is important that clear definitions are used throughout the system and across states. Following full implementation of SORMAS a detailed evaluation would improve understanding of any regional surveillance infrastructure issues or reporting biases. This may help explain some surveillance artefacts, such as high case numbers in some areas compared to others, that could affect interpretation of studies using this surveillance data.

Further understanding on an association with mortality and delayed presentation would be worthwhile, which would require complete follow-up information on mortality status following confirmed LF diagnosis.

To reduce residual confounding, further data could be collected on social and structural factors such as health literacy level, homelessness, poverty, physical disability, similar to McQuilkin et al., 2017 in Liberia during the Ebola epidemic [26]. This will improve knowledge on healthcare access issues and attitudes during outbreaks. This may be difficult to establish and collect in routine surveillance.

Further quantitative and qualitative studies should be considered beyond routinely collected surveillance and collect data underpinned by frameworks of delays, such as the three-delays model [14, 15]. Adewole et al., 2017 collected data on other determinants of poor health seeking behaviour such as out of pocket payment, health insurance coverage and levels of self-treatment [27]. Although Hulland et al. [22] have done work in investigating travel times to a healthcare facility, a useful enhancement would be to collect information on travel time from residence to healthcare facility, to assess accessibility.

It is important to recognise that risk factors may change with time, so where plausible surveillance should be updated to capture additional risk factors for investigation.

Once outbreak surveillance data quality is improved over a series of LF outbreak years, this study can be repeated to generate further hypotheses. This will allow for targeted public health interventions such as education and risk communication to reduce the mortality of confirmed LF cases.

## Conclusions

Whilst we observed important differences in presentation delays across factors, our study did not indicate a statistical association of any routinely collected factors with a delayed presentation of LF cases to a healthcare facility. Such associations may exist, but incomplete routine outbreak surveillance data may have hampered our ability to identify them. However, almost half of confirmed LF cases presented after 6 days of symptom onset, beyond the period in which ribavirin may be most effective. This highlights a need for improved national surveillance and studies to determine if there are systemic or specific causes for delays for LF and potentially other VHF cases. This information will help to inform, monitor, strengthen and evaluate risk communication and public health action strategies, to reduce the CFR associated with delayed care and treatment of LF cases.

## Data Availability

The data that support the findings of this study are available from Nigeria Centre for Disease Control but restrictions apply to the availability of these data, which were used under license for the current study, and so are not publicly available. Data are however available from the authors upon reasonable request and with permission of Nigeria Centre for Disease Control.

## Abbreviations

CFR: Case fatality rate
CI: Confidence Interval
IQR: Interquartile range
LF: Lassa fever
LGA: Local Government Area
NCDC: Nigeria Centre for Disease Control
RNA: Ribonucleic acid
SORMAS: Surveillance Outbreak Response Management and Analysis System
UK: United Kingdom
VHF: Viral haemorrhagic fever
